# Continuous performance tests in the assessment of children with attention-deficit/hyperactivity disorder: a narrative review

**DOI:** 10.3389/fnins.2026.1962306

**Published:** 2026-10-22

**Authors:** Yao Zhou, Xiaoke Zhao

**Affiliations:** Department of Rehabilitation, Children’s Hospital of Nanjing Medical University, Nanjing, China

**Keywords:** attention-deficit/hyperactivity disorder, continuous performance test, pediatric assessment, reaction time variability, technology-enhanced assessment

## Abstract

Attention-deficit/hyperactivity disorder (ADHD) is diagnosed primarily through clinical interviews, behavioral observations, and rating scales, while continuous performance tests (CPTs) provide complementary, performance-based measures of sustained attention, response inhibition, and response consistency. This narrative review compares major CPT paradigms used in pediatric ADHD assessment according to their cognitive targets, task characteristics, psychometric properties, ecological validity, clinical applications, and evidence maturity. Traditional standardized paradigms generally provide more established normative and psychometric frameworks, with the strongest support available for the Conners CPT series and TOVA, whereas distraction-enhanced, motion-integrated, virtual reality-based, and eye-tracking-integrated approaches extend assessment toward more complex and ecologically relevant behavioral contexts. Evidence from systematic reviews and meta-analyses supports group-level differences in core CPT measures, particularly reaction time variability, omission errors, and commission errors; however, substantial overlap across ADHD, psychiatric comorbidities, developmental stages, and other clinical conditions limits their specificity for individual diagnosis. Technology-enhanced paradigms may provide additional information regarding distractibility, motor activity, and gaze behavior, but their incremental clinical value remains uncertain because of heterogeneous methodologies, limited independent replication, and insufficient large-scale cross-cultural normative data. CPTs should therefore be interpreted as objective adjuncts within a multimodal assessment framework rather than as standalone diagnostic or subtype-classification tools. Future research should prioritize standardized outcome definitions, age-appropriate normative datasets, management of practice effects in repeated testing, and prospective multicenter studies evaluating whether specific CPT paradigms add clinically meaningful information beyond established diagnostic and treatment-monitoring procedures.

## 1 Introduction and background

Attention-deficit/hyperactivity disorder (ADHD) is one of the most common neurodevelopmental disorders in childhood, affecting approximately 5% of children worldwide (Polanczyk et al., 2007). According to the Diagnostic and Statistical Manual of Mental Disorders, Fifth Edition (DSM-5), ADHD is characterized by persistent symptoms of inattention and/or hyperactivity-impulsivity that impair academic, social, or emotional functioning (American Psychiatric Association, 2013). These symptoms are associated with impairments in attentional regulation, inhibitory control, and executive functioning, which have been linked to alterations in frontostriatal and frontoparietal networks (Barkley, 1997; Rubia, 2018; Willcutt et al., 2005). Current diagnosis relies primarily on clinical interviews, behavioral observations, and rating scales based on information obtained across settings (Wolraich et al., 2019). Although these approaches remain essential, their interpretation may be influenced by informant and contextual variability (Mitsis et al., 2000). Objective performance-based measures have therefore been investigated as complementary tools for characterizing ADHD-related cognitive functioning, but none has demonstrated sufficient specificity to replace comprehensive clinical evaluation (Arrondo et al., 2024). Among these measures, the continuous performance test (CPT) is widely used to assess sustained attention, response inhibition, and response consistency through indices such as omission errors, commission errors, and reaction-time variability (Hall et al., 2016; Kofler et al., 2013). CPT paradigms have evolved from traditional standardized tasks toward technology-enhanced approaches incorporating distraction, virtual reality, motion tracking, and eye tracking (Elbaum et al., 2020; Parsons, 2015; Yeh et al., 2020). However, substantial heterogeneity in task design, outcome measures, normative evidence, ecological validity, and clinical applicability limits direct comparison across paradigms. This narrative review therefore compares major CPT paradigms in pediatric ADHD assessment according to their cognitive targets, psychometric characteristics, ecological validity, clinical applications, and evidence maturity, while identifying current limitations and priorities for future research.

## 2 Literature search and review approach

This narrative review was informed by structured searches of PubMed/MEDLINE, Web of Science, Scopus, and PsycINFO for English-language publications from database inception to June 2026. Search terms combined descriptors of attention-deficit/hyperactivity disorder (“attention-deficit/hyperactivity disorder” OR “ADHD”) with terms relating to continuous performance testing (“continuous performance test” OR “CPT” OR “Conners CPT” OR “TOVA” OR “IVA-CPT” OR “MOXO-DCPT” OR “QbTest”) and technology-assisted assessment (“virtual reality” OR “motion tracking” OR “eye tracking” OR “machine learning”). Additional terms relating to psychometric properties, normative data, diagnostic accuracy, ecological validity, treatment monitoring, neurophysiology, and neuroimaging were used where appropriate. Reference lists of relevant reviews, meta-analyses, and key primary studies were also screened to identify additional publications. Eligible evidence included systematic reviews, meta-analyses, randomized controlled trials, prospective and retrospective cohort studies, diagnostic-accuracy and validation studies, cross-sectional studies, and seminal methodological publications relevant to CPT use in children and adolescents with ADHD. Adult studies were considered only when pediatric evidence was unavailable or when they provided important methodological information regarding emerging technologies. Studies examining attention tasks that did not meet the defining characteristics of a CPT, reports without ADHD-relevant outcomes, and publications lacking sufficient methodological detail were excluded. Official test manuals and manufacturer documentation were used only to verify platform-specific task procedures, scoring definitions, age ranges, and normative characteristics—that is, features of task administration, scoring, or normative data that are defined independently by each CPT manufacturer and are not necessarily standardized or interchangeable across different CPT systems—rather than to establish clinical efficacy. Evidence was appraised using a pragmatic hierarchy, with systematic reviews and meta-analyses given the greatest weight, followed by randomized or prospective multicenter studies, independent diagnostic and psychometric validation studies, observational cohort studies, and exploratory single-site or proof-of-concept investigations. Because the objective was to provide a conceptually structured narrative synthesis rather than a formal systematic review, no quantitative pooling or formal risk-of-bias assessment was undertaken. This approach was used to compare CPT paradigms according to their cognitive targets, task characteristics, psychometric support, ecological validity, clinical applications, limitations, and overall evidence maturity. For the qualitative evidence-maturity classification used in this review, comparatively mature paradigms were defined as those supported by established normative data, independent psychometric validation, and clinical-impact or review-level evidence. Developing paradigms had multiple validation studies but limited independent, cross-cultural, or clinical-impact evidence, whereas preliminary paradigms were supported mainly by small, single-site, or proof-of-concept investigations. Specifically, a paradigm was rated comparatively mature only when all three criteria were substantially met; it was rated preliminary when essentially none of the three criteria were met, with supporting evidence confined to isolated or proof-of-concept studies; and it was rated developing when the criteria were only partially or unevenly met—for example, when established normative data or multiple validation studies existed but independent replication or clinical-impact evidence remained limited or not yet demonstrated. These three criteria—normative data, independent replication, and clinical-impact evidence—were applied uniformly across all paradigm categories regardless of the underlying technology, so that, for example, a virtual-reality-based or eye-tracking-integrated paradigm was held to the same evidentiary standard as a traditional standardized CPT. This classification is intended as a qualitative appraisal of the evidence base for narrative purposes, rather than an exhaustive or quantitatively coded evidence inventory or a formal, quantitatively derived quality or risk-of-bias score; the evidence underlying each paradigm’s classification—including study designs, independent replication status, available normative datasets, and diagnostic or clinical-impact evidence—is summarized in Supplementary Table S2.

## 3 Cognitive framework and neural basis of CPT performance

The Continuous Performance Test (CPT) was originally developed as a vigilance task requiring sustained monitoring over time (Rosvold et al., 1956). Its later application in ADHD reflected a broader shift from viewing the disorder primarily in terms of motor overactivity toward recognizing difficulties in attentional regulation and inhibitory control (Albrecht et al., 2015; Douglas, 1972; Douglas and Peters, 1979). CPTs subsequently evolved from relatively simple vigilance tasks into performance-based paradigms sampling several attentional and executive processes (Riccio, 2002).

For the purposes of this review, CPT performance is organized into three interrelated cognitive domains: sustained attention, response inhibition, and response consistency. Sustained attention refers to the capacity to maintain goal-directed task engagement and detect infrequent target stimuli over an extended period, and is commonly indexed by omission errors (Oken et al., 2006; Sarter et al., 2001). This construct is related to, but conceptually distinct from, vigilance and tonic alertness: although these processes rely on overlapping fronto-parietal and ascending arousal systems, alertness denotes a general state of physiological readiness, whereas sustained attention denotes the top-down, goal-directed maintenance of engagement over time (Oken et al., 2006).

Response consistency, commonly assessed through reaction-time variability (RTV), reflects trial-to-trial fluctuations in performance. Although omission errors and RTV are both frequently treated in the literature as indices of sustained attention, this review follows cognitive-energetic and state-regulation accounts of ADHD, which propose that RTV more specifically reflects fluctuations in energetic state and effortful control allocation across the task, whereas omission errors more directly reflect the overall level of task engagement (Castellanos and Tannock, 2002; Sergeant, 2000). This review therefore treats response consistency as a partially separable construct for conceptual clarity, while acknowledging that the two indices are empirically correlated and are not treated as fully independent constructs in all prior work.

These three constructs also map onto complementary theoretical accounts of ADHD, which together help explain why CPT-derived measures—particularly RTV—are sensitive to the disorder. Barkley's (1997) influential model frames ADHD primarily as a disorder of behavioral inhibition, from which secondary impairments in sustained attention and executive control follow; this account maps most directly onto response inhibition as indexed by commission errors. The cognitive-energetic model (Sergeant, 2000) and related state-regulation accounts (Castellanos and Tannock, 2002) offer a complementary explanation more specific to response consistency: rather than treating ADHD-related performance problems as a fixed capacity deficit, these models propose that ADHD involves impaired regulation of energetic state—arousal, activation, and effort—across the course of a task, such that performance becomes unstable from moment to moment rather than uniformly impaired throughout. Under this account, elevated RTV does not simply index a generalized ‘sustained attention deficit’ but more specifically reflects fluctuating attentional engagement and energetic-state regulation over time—a distinction consistent with the vigilance/alertness/sustained-attention framework introduced above (Oken et al., 2006). Because CPTs sample performance continuously across an extended task duration (see below), they are particularly well positioned to detect this kind of within-task instability, which may explain why RTV has emerged as one of the most consistently replicated group-level findings in ADHD across the literature, identified in a meta-analysis spanning 319 studies (Kofler et al., 2013).

This state-regulation framing also helps reconcile an apparent paradox in the CPT literature: robust, well-replicated group-level differences in RTV and related indices coexist with modest diagnostic accuracy at the level of the individual child (see Section 7.1). If elevated RTV reflected a relatively stable, trait-like attentional deficit, one would expect it to discriminate individual cases more reliably than it does. If, instead, RTV reflects moment-to-moment fluctuations in energetic state that are themselves modulated by motivation, incentive, arousal, and situational context, then a single testing session captures only one sample of an inherently variable process; large-sample designs can detect the resulting average group difference with considerable statistical power, while any one child’s performance on a given occasion may or may not fall within the range typical of that fluctuation. This account does not resolve the diagnostic-specificity limitations discussed later in this review, but it offers a theoretically grounded explanation for why they occur, rather than treating modest individual-level accuracy as simply a psychometric shortcoming of existing CPT platforms.

These cognitive domains are assessed within a task structure defined by parameters such as event rate, the inter-stimulus interval (ISI) — the interval between successive stimulus presentations, which may be fixed, block-varied, or adaptively adjusted according to the subject’s ongoing accuracy — and overall task duration. Consistent with Rosvold et al.'s (1956) original criteria, a CPT is characterized by the presentation of constantly changing stimuli with a target occurring at low frequency relative to the total number of stimuli presented over an extended duration, a structure intended to sample sustained monitoring over time (Riccio, 2002). This combination of a controlled ISI, low target frequency, and extended task duration is what allows CPTs to sample sustained attention as the capacity to maintain focus on infrequent critical events over time. Target infrequency alone, however, does not cleanly distinguish CPTs from go/no-go tasks: because traditional go/no-go tasks also typically embed relatively rare no-go stimuli, correct performance on such tasks likewise depends in part on sustained attentional engagement, and this conceptual overlap is precisely why factorial designs manipulating stimulus frequency independently of response type have been used to dissociate sustained attention from response inhibition (Hwang et al., 2019). The extended administration period over which CPTs maintain a controlled ISI and a continuous stimulus stream — intended to induce a vigilance decrement over time — therefore represents a more specific feature distinguishing CPTs from the comparatively brief administration typical of standard go/no-go tasks. ISI length also appears to interact with the cognitive domains outlined above and with the neural response to stimuli: shorter ISIs have been associated with greater sensitivity to attentional dysfunction and with changes in P3 latency and amplitude (Riccio, 2002). Because ISI and event rate differ across CPT platforms and versions, comparisons of omission, commission, and RTV findings across studies should take these task-timing parameters into account rather than assuming equivalent task demands.

These processes depend on distributed neural systems rather than a single brain region, spanning frontoparietal, frontostriatal, and salience-network circuits involved, respectively, in sustained attentional control, response selection and inhibition, and the detection of behaviorally relevant events (Castellanos and Proal, 2012; Hart et al., 2013). Because these same networks are implicated across multiple psychiatric and neurodevelopmental conditions rather than being specific to ADHD, the neural substrates of CPT performance are best understood as distributed and transdiagnostic (McTeague et al., 2017; Rubia, 2018)—a property with direct implications for diagnostic specificity that this review returns to in Section 7.1, where the overlap between ADHD and autism spectrum disorder is examined in detail.

## 4 Traditional CPT performance measures

Traditional CPTs, including the Conners CPT series, TOVA, and IVA-CPT, share a core set of performance measures used to characterize the three cognitive domains outlined above—sustained attention, response inhibition, and response consistency—alongside basic response speed, indexed by mean reaction time (RT). Omission errors refer to failures to respond to target stimuli and are commonly interpreted as lapses in sustained attention, whereas commission errors are inappropriate responses to non-target stimuli and may reflect reduced response inhibition or a relatively liberal response strategy (Epstein et al., 2003; Losier et al., 1996). Mean reaction time (RT) reflects average response speed, while reaction time standard deviation (RT SD) describes the dispersion of response times across trials. In the Conners CPT-3, perseverations are defined as responses occurring less than 100 ms after stimulus presentation and may indicate anticipatory, random, or highly impulsive responding (Conners, 2014).

RTV warrants particular emphasis because it captures intraindividual fluctuations that may not be apparent from mean RT alone. Depending on the task and analytical approach, it may be operationalized using RT SD, the coefficient of variation, ex-Gaussian parameters such as τ, or trial-level analyses (Antonini et al., 2013; Tamm et al., 2025). Even among the traditional platforms summarized in Table 1, RTV-related scoring is not uniform: the Conners CPT-3 reports both an overall Hit RT Standard Deviation and a separately calculated, block-based Variability index, whereas TOVA reports a single Response Time Variability score reflecting the consistency of correct response times (Conners, 2014). Because these platform-specific indices are not numerically interchangeable, comparisons of ‘RT SD/RTV’ findings across traditional CPT platforms should specify which index is being referenced. As discussed in Section 3, RTV is among the most consistently replicated group-level findings in ADHD (Kofler et al., 2013). Substantial overlap with other clinical populations, however, limits its diagnostic specificity.

**Table 1:** Comparative characteristics and evidence maturity of major CPT paradigms in pediatric ADHD.

Dimension	Traditional visual CPTs: Conners CPT-3, TOVA	Cross-modal CPT: IVA-2	Distraction-enhanced CPT: MOXO-DCPT	Motion-integrated CPT: QbTest	Motion-response CPT: Kinect-CPT	VR classroom CPTs: AULA/Nesplora, ClinicaVR	Eye-tracking/multimodal CPTs
Task design	Structured visual target-detection/go–no-go tasks	Integrated visual–auditory target detection	Target-detection CPT with visual and auditory distractors	CPT with infrared motion tracking	Dominant-hand responses tracked by Kinect	CPT in a simulated classroom with controlled distractors	CPT combined with eye tracking, sensors, or machine learning
Cognitive/behavioral domains targeted	Sustained attention; response inhibition; response consistency; response speed (reference baseline for the columns to the right)	Shared with traditional CPTs: sustained attention, response inhibition, response consistency, response speed — assessed across visual and auditory modalities	Shared with traditional CPTs: sustained attention, response inhibition. Additional: behavioral regulation under controlled environmental distraction	Shared with traditional CPTs: sustained attention, response inhibition, response consistency. Additional: motor activity/behavioral regulation via infrared motion tracking	Shared with traditional CPTs: response inhibition, response speed. Additional: response execution via tracked hand movement, replacing manual button-press responses	Shared with traditional CPTs: sustained attention, response inhibition. Additional: context-dependent distractibility and behavioral regulation under simulated classroom conditions	Shared with the underlying CPT platform: sustained attention, response inhibition. Additional: visual attentional allocation and disengagement, off-task behavior, via eye tracking
Key measured outcomes	Omissions; commissions; RT; RT variability (Conners CPT-3: Hit RT Standard Deviation plus a separately reported block-based Variability index; TOVA: a single Response Time Variability index); detectability (d′); response style (β)	FSAQ; FSRCQ; Vigilance; Focus; Speed; Prudence; Consistency; Stamina	Attention; Timing; Impulsivity; Hyperactivity	Omissions; commissions; RT; RTV; motion-derived activity measures	RT; commission errors; inhibited responses; action-completion time	Conventional CPT indices; head movement/orientation; gaze measures	Fixation; dwell time; gaze allocation; saccades; off-task gaze; CPT indices
Normative evidence	Large, platform-specific standardization samples	Age- and sex-referenced norms	Pediatric normative data available	Age- and sex-referenced norms	No large normative database established	Normative coverage varies considerably across platforms	No shared or established normative dataset
Psychometric evidence	Established reliability and validity support	Less independent psychometric validation than major visual CPTs	Limited independent and cross-cultural replication	Regulatory clearance; supported by review-level and randomized clinical-impact evidence	Limited to a single small proof-of-concept study; no independent replication	Meta-analytic group-level evidence; psychometric support and cross-cultural validation vary across systems	Mostly small, single-site pilot studies; no standardized outcome definitions
Ecological resemblance	Low to moderate	Low to moderate	Moderate	Moderate	Moderate	Relatively high	Task-dependent
Dimension	Traditional visual CPTs: Conners CPT-3, TOVA	Cross-modal CPT: IVA-2	Distraction-enhanced CPT: MOXO-DCPT	Motion-integrated CPT: QbTest	Motion-response CPT: Kinect-CPT	VR classroom CPTs: AULA/Nesplora, ClinicaVR	Eye-tracking/multimodal CPTs
Clinical application	Multimodal assessment; treatment monitoring	Visual–auditory attention and response-control profiling	Assessment of context-dependent distractibility	Clinical assessment and assessment-efficiency support	Research	Assessment of classroom-like distractibility	Investigational research
Key limitations	Limited ecological representation; modest diagnostic specificity; limited cross-platform comparability	Proprietary composites; auditory/language demands; limited cross-platform comparability	Incremental utility uncertain; sensitive to distractor design and task context	Modest diagnostic accuracy; proprietary hardware, scoring, and norms	Hand movement does not represent general hyperactivity; device dependence; limited validation	Ecological realism does not ensure diagnostic superiority; platform heterogeneity	Device, calibration, and algorithm dependence; heterogeneous metrics; limited replication
Evidence maturity	Comparatively mature	Developing	Developing	Comparatively mature	Preliminary	Developing	Preliminary

Evidence-maturity tiers (comparatively mature / developing / preliminary) follow the criteria defined in Section 2 — normative data, independent replication, and clinical-impact evidence — applied uniformly across paradigms; the supporting evidence for each rating is detailed in Supplementary Table S2. Ecological resemblance is a qualitative judgment (not a validated psychometric scale) reflecting how closely a task’s surface features approximate real-world attentional and behavioral demands, ranging from structured, decontextualized laboratory tasks (low) through tasks incorporating controlled distraction or motor-response demands (moderate) to tasks embedded in simulated real-world contexts such as a virtual classroom (relatively high); “task-dependent” indicates that resemblance depends on the specific underlying CPT platform to which eye-tracking or other sensors were added. This rating describes resemblance and experimental complexity only, not demonstrated ecological validity: for no paradigm summarized here, including virtual-reality and distraction-enhanced systems, is there currently sufficient direct evidence that test performance predicts classroom behavior, academic functioning, or everyday attentional/functional impairment more accurately than traditional CPT performance (see Section 7.4). “Cognitive/behavioral domains targeted” lists the constructs each paradigm is intended to assess, framed relative to the traditional-CPT baseline in the first column; “Key measured outcomes” lists the specific, platform-defined indices through which those constructs are operationalized and should not be assumed numerically interchangeable across platforms. RT, reaction time; RTV, reaction time variability. RT SD and RTV are not uniformly reported across platforms: for example, Conners CPT-3 reports both an overall Hit RT Standard Deviation and a separate block-based Variability index, whereas TOVA reports a single Response Time Variability index; alternative RTV operationalizations (e.g., coefficient of variation, ex-Gaussian parameters) are discussed narratively in Section 4. FSAQ, Full-Scale Attention Quotient; FSRCQ, Full-Scale Response Control Quotient.

### 4.1 Signal detection theory indices

Signal detection theory provides complementary interpretive information by separating perceptual sensitivity from response strategy. The sensitivity index (d′) reflects an individual’s ability to discriminate target stimuli from non-target stimuli, whereas response bias, commonly represented by β or criterion c, reflects the tendency to adopt a relatively liberal or conservative response criterion (Stanislaw and Todorov, 1999). Meta-analytic evidence suggests that children with ADHD generally show reduced perceptual sensitivity compared with typically developing children, while findings regarding response bias are less consistent (Huang-Pollock et al., 2012). Although d′ and response-bias measures are associated with ADHD-related performance, they do not demonstrate sufficient specificity for distinguishing inattentive from hyperactive–impulsive symptom dimensions (Epstein et al., 2003).

Omission errors, commission errors, RT, RT SD, RTV, perseverations, d′, and response-bias measures should therefore be interpreted jointly and in relation to the broader clinical context rather than as isolated indicators (Hall et al., 2016).

## 5 Taxonomy of CPT paradigms: from standardized tasks to technology-enhanced assessment

Rather than representing competing diagnostic instruments, CPT paradigms have progressively evolved to address different clinical and methodological challenges, ranging from the standardized measurement of attentional control to more ecologically oriented assessments intended to approximate real-world behavioral regulation (Albrecht et al., 2015; Riccio, 2002). Over recent decades, CPT development has expanded from traditional laboratory-based paradigms focusing on sustained attention and inhibitory control toward approaches incorporating controlled environmental distraction, motion monitoring, virtual reality, and eye-tracking-based data acquisition (Berger et al., 2017; Delgado-Gomez et al., 2017; Parsons et al., 2019; Stokes et al., 2022). These paradigms emphasize different aspects of cognitive and behavioral performance and should not be assumed to be psychometrically interchangeable (Arrondo et al., 2024). For the purposes of this review, current CPT paradigms are organized into five groups according to their methodological characteristics and clinical objectives: traditional standardized CPTs, distraction-enhanced CPTs, motion-integrated CPTs, virtual reality-based CPTs, and emerging multimodal technology-assisted CPTs. The comparative characteristics and evidence maturity of these paradigms are summarized in Table 1, while platform-specific technical specifications are provided in Supplementary Table S1. When evaluating each paradigm below, it is important to distinguish between levels of evidence that are sometimes conflated in the CPT literature. A statistically significant group-level difference between children with ADHD and a comparison group indicates only that average scores differ; it does not by itself establish clinically useful diagnostic accuracy, which requires reporting of sensitivity, specificity, positive and negative predictive values, and likelihood ratios at clinically relevant score thresholds. Nor does diagnostic accuracy alone establish incremental validity—that is, whether a paradigm adds clinically meaningful information beyond what is already available from standard clinical interviews, behavioral rating scales, and observation, which is ultimately the more clinically relevant question (Epstein and Weiss, 2012). This distinction is particularly important for newer, more technologically complex paradigms: as discussed below, much of the evidence currently available for motion-tracking, virtual-reality, eye-tracking, and machine-learning-based approaches consists of group-level classification or group-difference findings rather than the sensitivity/specificity, predictive-value, or incremental-validity evidence needed to justify their use in individual clinical decision-making, and the sophistication of a measurement approach should not be assumed, on its own, to translate into greater clinical utility.

### 5.1 Traditional standardized CPT paradigms

Traditional standardized CPT paradigms, including the Conners CPT series, TOVA, and IVA-CPT, use highly structured target-detection or response-inhibition tasks administered under controlled testing conditions. The Conners CPT and TOVA primarily use visual stimuli, whereas the IVA-CPT integrates visual and auditory targets and non-targets to assess performance across sensory modalities (Greenberg and Waldmant, 1993; Tinius, 2003). These paradigms primarily assess sustained attention, response inhibition, response speed, and response consistency through outcomes such as omission and commission errors, mean reaction time, reaction time variability, detectability, response style, and platform-specific indices of attention and response control (Conners, 2014; Homack and Riccio, 2006). Beyond these core measures, the IVA-CPT and IVA-2 also generate platform-specific composite scores, including the Full-Scale Attention Quotient and Full-Scale Response Control Quotient, together with auditory and visual scales assessing Vigilance, Focus, Speed, Prudence, Consistency, and Stamina (Sandford, 2014; Tinius, 2003). Here, the IVA-specific “Vigilance” score is treated as a platform-specific composite index rather than as a separate cognitive domain in this review. These composite scores summarize performance across multiple response dimensions and sensory modalities using IVA-specific definitions and age-normed transformations; for example, IVA-2 Focus and Consistency-related scores should not be assumed numerically interchangeable with RT SD, coefficient-of-variation measures, or RTV scores derived from other CPT platforms (Sandford, 2014). Compared with newer CPT approaches, traditional standardized paradigms generally have more established administration procedures and normative frameworks, although the breadth and quality of independent psychometric evidence vary across platforms. Their ecological validity is comparatively limited because performance is measured in quiet, highly controlled environments that do not reproduce the distractors, motivational demands, and contextual variability of everyday functioning (Parsons, 2015). In clinical practice, these paradigms are commonly used to characterize attentional performance, support multimodal ADHD assessment, and, in some settings, monitor treatment-related change. Their main limitations include modest diagnostic specificity, restricted representation of everyday behavior, and incomplete comparability of similarly named indices across platforms. Nevertheless, traditional standardized CPTs currently have the most mature normative and psychometric foundations among the paradigms reviewed.

### 5.2 Distraction-enhanced CPT paradigms

Distraction-enhanced CPT paradigms, primarily represented by the MOXO-dCPT, retain a computerized target-detection structure while incorporating controlled visual, auditory, and combined environmental distractors (Cassuto et al., 2013). These tasks are designed to assess attentional performance, response control, and behavioral regulation under distracting conditions and generate four platform-specific indices: Attention, reflecting correct responses to target stimuli; Timing, which distinguishes responses produced within the designated response window from delayed correct responses; Impulsivity, reflecting responses to non-target stimuli; and Hyperactivity, capturing excessive, repeated, or random key presses occurring during the task (Berger et al., 2017; Cassuto et al., 2013). These indices provide a multidimensional profile of performance under distraction but should not be interpreted as direct equivalents of similarly named clinical symptoms.

Platform-specific validation studies indicate that the MOXO-dCPT can detect attentional and behavioral differences in pediatric samples (Rivella et al., 2024). By introducing controlled environmental interference, distraction-enhanced paradigms increase experimental complexity relative to traditional CPTs and may provide additional information about context-dependent distractibility; this added complexity may be particularly relevant when susceptibility to environmental distraction is central to the referral question, but it does not by itself establish that MOXO-dCPT performance predicts real-world distractibility more accurately than traditional CPT performance. However, performance can vary according to distractor modality, configuration, and task context (Berger and Cassuto, 2014).

Independent replication and evidence of incremental clinical utility remain less extensive than for traditional standardized CPTs, particularly regarding whether distraction-related indices improve diagnostic discrimination (e.g., sensitivity, specificity) or clinical decision-making beyond existing procedures. Distraction-enhanced CPTs therefore currently occupy a developing evidence tier.

### 5.3 Motion-integrated CPT paradigms

Motion-integrated CPT paradigms combine computerized cognitive tasks with objective recording of motor activity or response execution. QbTest integrates a computerized attention and impulse-control task with infrared motion tracking, whereas Kinect-based CPTs record dominant-hand movements used to respond to target stimuli rather than unrestricted whole-body activity. These paradigms assess sustained attention, response inhibition, reaction-time consistency, motor activity, and movement-related response execution. QbTest generates conventional outcomes such as omission errors, commission errors, reaction time, and reaction time variability together with motion-derived activity measures, whereas Kinect-based paradigms assess response initiation, reaction time, commission errors, inhibited responses, and action-completion time—measures that describe response preparation and execution rather than unrestricted whole-body hyperactivity (Bellato et al., 2024; Delgado-Gomez et al., 2017). Normative and psychometric evidence differs substantially within this category. QbTest uses age- and sex-referenced normative comparisons, has received regulatory clearance for use as an aid in ADHD assessment, and is supported by systematic-review, meta-analytic, and randomized clinical-impact evidence (U. S. Food and Drug Administration, 2012). In contrast, the evidence base for Kinect-based CPTs remains preliminary and is largely derived from a small proof-of-concept pediatric study without established large-scale normative or independent validation. The addition of movement measurement broadens behavioral coverage by capturing activity or response-execution data not represented by manual-response indices alone, although testing remains highly structured. In clinical settings, QbTest may support diagnostic decision-making and improve assessment efficiency, whereas Kinect-based approaches remain primarily research tools (Hollis et al., 2018). Important limitations of QbTest include proprietary hardware and scoring procedures and modest diagnostic discrimination across heterogeneous clinical samples, while Kinect-based measures should not be interpreted as indicators of unrestricted or generalized hyperactivity. QbTest is therefore comparatively mature among technology-assisted CPTs, whereas Kinect-based CPTs remain preliminary.

### 5.4 Virtual reality-based CPT paradigms

Virtual reality-based CPT paradigms, including AULA Nesplora and ClinicaVR, embed target-detection tasks within simulated classroom environments containing controlled visual, auditory, and contextual distractors (Iriarte et al., 2016; Neguț et al., 2017). These paradigms assess context-dependent sustained attention, distractibility, response inhibition, behavioral regulation, and, in some systems, motor activity or attentional orientation within the virtual environment. Their outcomes include conventional omission, commission, reaction-time, and variability measures together with system-specific measures such as head movement, orientation, gaze allocation, off-task gaze, and responses to environmental distractors (Yeh et al., 2020). Head-movement measures may specifically quantify the frequency, magnitude, and direction of movement, including pitch, yaw, and roll, and task-irrelevant head movement has been associated with ADHD symptom severity in preliminary pediatric research (Cho et al., 2022).

Meta-analytic and review-level evidence indicates that virtual-classroom CPTs can detect group-level differences between children with ADHD and comparison groups, although normative coverage, psychometric support, task design, and cross-cultural validation vary considerably across systems and populations (Areces et al., 2025; Goharinejad et al., 2022). Their principal advantage lies in greater ecological resemblance and experimental complexity—assessing performance under controlled, classroom-like distraction—which may be relevant when context-dependent inattention is central to the referral question. However, this resemblance to real-world settings has not been matched by demonstrated ecological validity: few studies have directly tested whether VR-CPT performance predicts classroom behavior, academic functioning, or everyday attentional difficulties more accurately than traditional CPT performance (see Section 7.4). Nor has this increased contextual complexity consistently produced superior diagnostic performance—in terms of sensitivity, specificity, or incremental validity over traditional CPTs—and differences in hardware, distractor configurations, outcome definitions, and normative procedures limit comparison across platforms (Arrondo et al., 2024). Neither greater ecological resemblance nor greater experimental complexity should, on its own, be assumed to confer demonstrated ecological validity or greater clinical utility.

### 5.5 Emerging multimodal technology-assisted CPTs

Emerging multimodal CPT paradigms combine conventional or virtual CPTs with eye tracking, behavioral sensors, or machine-learning analysis. Their purpose is to capture attentional allocation, distraction, off-task behavior, and cognitive–behavioral patterns that may not be evident from response accuracy and reaction time alone. Eye-tracking-integrated CPTs specifically assess fixation duration, dwell time, gaze allocation, viewing of distractors, saccadic behavior, gaze variability, and off-task gaze; these outcomes indicate where visual attention is directed, how readily attention disengages from task-relevant stimuli, and how environmental distractors influence performance (Elbaum et al., 2020; Lee et al., 2023).

Eye-tracking studies integrating the MOXO-dCPT have reported greater visual attention to irrelevant regions in adults with ADHD and possible improvements in group classification when gaze measures were added to conventional CPT indices (Lev et al., 2022). Pediatric studies have similarly identified atypical gaze allocation and off-task behavior during conventional or virtual-classroom CPTs (Lee et al., 2023; Stokes et al., 2022). Machine-learning models using CPT-derived or eye-tracking data have also produced promising classification results (Slobodin et al., 2020; Yoo et al., 2024).

These approaches may provide a broader representation of attentional regulation by combining discrete task responses with continuous behavioral data. However, the available evidence remains preliminary: reported findings largely reflect improved group-level classification accuracy in individual studies rather than externally validated sensitivity, specificity, or predictive values, and none has yet demonstrated incremental validity over standard clinical assessment. Improved group classification in individual studies has therefore not yet established routine clinical value, and the added measurement complexity of combining eye tracking or machine-learning analysis with conventional CPT indices should not be assumed, on its own, to translate into greater diagnostic or clinical usefulness. Eye-tracking-integrated and machine-learning-assisted CPTs should therefore currently be regarded as investigational tools for cognitive profiling and candidate-biomarker research rather than established clinical instruments.

Across these paradigms, indices with similar labels should not be assumed equivalent. Platform-specific composite scores—such as the IVA quotients and scales, or the MOXO-dCPT’s Attention, Timing, Impulsivity, and Hyperactivity indices—summarize performance using system-specific definitions and normative transformations, and should not be treated as numerically interchangeable with RT SD, coefficient-of-variation measures, or RTV scores derived from other platforms. Similarly, technology-derived measures such as head movement, hand movement, and gaze behavior should not be interpreted as alternative estimates of conventional reaction-time variability: RT SD reflects response-time dispersion and the coefficient of variation adjusts this dispersion for mean RT, whereas movement- and gaze-based measures instead capture motor execution, attentional orientation, and visual allocation (Tamm et al., 2012). Rather than offering alternative estimates of response accuracy or reaction-time variability, these technology-derived measures extend conventional CPT assessment by capturing the behavioral orientation, motor engagement, and environmental interaction that accompany task performance—a shift from what a child answered toward how attention and behavior unfold during the task, and the principal added value of technology-assisted CPTs relative to traditional paradigms.

Overall, traditional standardized CPTs retain the most developed normative and psychometric foundations, while QbTest has comparatively stronger evidence among technology-assisted systems (Bellato et al., 2024). Distraction-enhanced and VR-based paradigms remain developing, whereas eye-tracking-integrated, Kinect-based, and machine-learning-assisted approaches are primarily investigational.

## 6 Clinical interpretation and applications of CPT

### 6.1 Clinical value of CPTs combined with neurophysiological and neuroimaging measures

Combining CPTs with electrophysiological measures derived from electroencephalography (EEG)—principally quantitative EEG (qEEG) and event-related potentials (ERPs)—or with functional magnetic resonance imaging (fMRI) has primarily supported investigation of ADHD-related neural mechanisms rather than the development of clinically validated diagnostic biomarkers. Although qEEG and ERPs are both derived from the same underlying EEG recording, they capture different aspects of electrophysiological activity: qEEG characterizes the ongoing spectral or power-based properties of the resting or task-related EEG signal, whereas ERPs reflect the time-locked, stimulus-evoked electrical response averaged across trials. Reviews of ERP research have identified group-level alterations in components related to attentional allocation, inhibitory control, and performance monitoring, including reduced or delayed P3-related responses. However, findings vary across tasks, samples, and analytical procedures, and no ERP measure has shown sufficient reliability or specificity for individual diagnosis (Barry et al., 2003; Johnstone et al., 2013).

Studies combining CPT performance with qEEG measures have reported abnormalities in both CPT and qEEG indices considered separately, but evidence of incremental diagnostic value from combining them remains limited. For example, Kim et al. found differences between ADHD and control groups in IVA-CPT and quantitative EEG measures, but did not demonstrate that combining them improved diagnostic performance beyond either modality alone (Kim et al., 2015).

Task-based fMRI studies likewise identify group-level differences in networks involved in attention, cognitive control, response inhibition, and default-mode processing (Cortese et al., 2012). These findings support altered neural functioning in ADHD but do not provide an individually reliable or disorder-specific diagnostic marker.

Accordingly, CPT-qEEG, CPT-ERP, and CPT-fMRI combinations should currently be regarded as exploratory approaches for mechanistic research and candidate-biomarker development. Large prospective multicenter studies using prespecified multimodal models, external validation, and comparison with standard clinical assessment are required to determine whether these combinations provide clinically meaningful incremental diagnostic information.

### 6.2 Clinical interpretation and comorbidities

An abnormal CPT result should be interpreted through a structured, stepwise process rather than on the basis of a single score or cutoff. First, clinicians should determine whether the result is interpretable by reviewing the child’s comprehension of the instructions, language and sensory abilities, cooperation and task engagement, any validity indicators available for the specific CPT, medication status, sleep, fatigue, and emotional state at the time of testing. Results obtained during marked anxiety, insufficient sleep, poor engagement, or misunderstanding of the instructions should be interpreted cautiously and, when clinically necessary, reassessed under more stable conditions (Areces et al., 2021; Gruber et al., 2007).

Second, clinicians should examine the configuration and temporal course of performance across multiple indices and task blocks. Elevated omission errors accompanied by marked reaction time variability may be consistent with intermittent lapses in task engagement, although neither finding is specific to ADHD (Huang-Pollock et al., 2012; Kofler et al., 2013). In contrast, uniformly slow but relatively stable responding may warrant consideration of generalized processing-speed or broader cognitive limitations rather than being attributed specifically to ADHD. Commission errors should likewise be interpreted in relation to reaction time, response bias, target frequency, and the conditions under which they occur, rather than being treated automatically as evidence of impulsivity (Conners, 2014).

Third, CPT findings should be interpreted in relation to the child’s developmental level, intellectual functioning, cognitive and language profile, comorbid conditions, and symptoms reported across settings. Intellectual functioning can influence reaction time, reaction time variability, omission errors, and commission errors, while state anxiety may also affect CPT performance and complicate disorder-specific interpretation (Hurford et al., 2017; Puga et al., 2019).

Finally, clinicians should determine whether the observed difficulties are persistent, developmentally inappropriate, functionally impairing, and consistent with information obtained from multiple informants across home and school settings. When CPT abnormalities occur only under particular testing conditions, are inconsistent with everyday functioning, or are better explained by anxiety, sleep disturbance, language or learning difficulties, intellectual disability, medication effects, or another comorbid condition, they should be given less weight as evidence for ADHD. CPT data are therefore most clinically useful as an adjunct to comprehensive assessment, helping to refine the differential formulation and identify cognitive processes requiring further evaluation, rather than serving as an independent diagnostic test (Wolraich et al., 2019).

### 6.3 Developmental considerations

CPT interpretation should account for age-related variation in the development of sustained attention, inhibitory control, and response consistency. Cognitive control abilities continue to improve substantially from early childhood through adolescence (Davidson et al., 2006), and CPT indices such as omission errors, commission errors, reaction time, and response variability show marked developmental changes across age groups (Tsushima et al., 2010). Consequently, poorer or more variable CPT performance in children aged 4–6 years may partly reflect normative developmental variability rather than ADHD-specific dysfunction.

Studies of preschool children further indicate that CPT error patterns are complex, may change over the course of the task, and are influenced by multiple behavioral factors, limiting interpretation based on a single cutoff or index (Allan and Lonigan, 2015; Kerns and Rondeau, 1998). Age-appropriate task design and normative data are therefore essential. Preschool-specific CPTs have been developed using shorter and developmentally adapted procedures, and some commercial tests apply different versions or norms to younger children. For example, the original TOVA study reported developmental normative data for children aged 6–16 years, whereas the current professional manual specifies a shortened visual version and separate norms for 4- and 5-year-olds; the full 21.6-min visual test is normed from age 6 onward (Greenberg and Waldmant, 1993; Leark et al., 2020).

Clinicians should therefore verify that the task version and normative sample adequately represent the child’s age band and interpret preschool CPT findings together with developmental history, behavioral observations, cognitive and language abilities, and functional impairment. Applying norms or task demands developed for older children to preschool populations may otherwise increase the risk of developmental misclassification.

### 6.4 Differentiating ADHD symptom profiles and cognitive profiles

CPT performance patterns show some associations with ADHD symptom dimensions; however, these findings should be interpreted as group-level tendencies rather than reliable indicators for individual presentation classification. Inattentive symptoms have been associated with increased omission errors and slower or more variable responding in some cohorts, whereas commission errors are commonly interpreted as reflecting reduced response inhibition. Nevertheless, large-sample evidence indicates that individual CPT measures do not map consistently or specifically onto inattentive versus hyperactive–impulsive symptom domains (Epstein et al., 2003; López-Vicente et al., 2014). Studies comparing predominantly inattentive and combined presentations have identified some neurocognitive differences, but substantial within-group variability and between-group overlap remain (Solanto et al., 2007).

Technology-enhanced CPT paradigms may capture additional symptom-related differences under more complex conditions. Studies using the AULA virtual classroom reported differences among ADHD presentations and, in specific samples, greater discriminative performance than TOVA; however, these findings derive from individual cross-sectional studies and require independent replication (Areces et al., 2018; Rodríguez et al., 2018). Similarly, MOXO-DCPT parameters have been associated with internalizing and externalizing symptoms in children with ADHD, but this evidence reflects correlational relationships rather than validated subtype classification (Öztürk, 2023).

Overall, CPT-derived patterns may support cognitive profiling but are not sufficiently reliable for individual presentation classification. Prospective, multicenter, and independently validated studies are needed to determine whether symptom-guided selection of CPT paradigms provides meaningful incremental clinical value beyond established diagnostic procedures.

### 6.5 Treatment monitoring and rehabilitation applications

Compared with their diagnostic use, CPTs have been studied more directly as measures of treatment-related cognitive change and show some sensitivity to pharmacological effects, although their clinical value varies across platforms, indices, and study designs (Epstein and Weiss, 2012). In this context, it is important to distinguish among three related but conceptually distinct levels of change: change in CPT-measured cognitive performance (e.g., reaction-time variability, omission errors, response accuracy), change in clinically rated symptom severity, and change in real-world functional outcomes such as academic, family, or social functioning. CPTs can quantify changes in attentional stability, response inhibition, response speed, and response consistency, but a statistically detectable change on these cognitive indices does not necessarily correspond to a meaningful change in symptom severity or functional outcomes, which are shaped by additional factors beyond task performance. CPT-derived measures should therefore complement rather than replace symptom ratings, functional assessments, and clinician-based evaluations.

Stimulants, particularly methylphenidate, have shown the most consistent effects on CPT performance, including reductions in omission and commission errors and possible reductions in reaction-time variability (Losier et al., 1996; Riccio et al., 2001). In a small double-blind crossover study, VR-CPT, TOVA, and a non-VR CPT were all sensitive to methylphenidate-related changes, with particularly marked improvement in VR-CPT omission errors (Pollak et al., 2010). Atomoxetine and methylphenidate have also been compared directly: Bédard et al. reported that methylphenidate produced greater improvements than atomoxetine across several CPT measures, including reaction time, reaction-time variability, and omission errors—findings the original study described broadly as improvements in sustained attention, using a composite construct that does not map directly onto the sustained attention/response consistency distinction adopted in Section 3 of this review (Bédard et al., 2015). Evidence regarding other pharmacological agents remains limited. Repeated administration requires consideration of test–retest reliability, measurement error, practice effects, and retest interval. These influences are platform- and population-specific: repeated TOVA administration showed no significant practice effects in adult ADHD trial samples, but this finding may not generalize to children or other CPT systems (Rotem et al., 2019). Where appropriate reliability and normative data are available, reliable change indices may help distinguish meaningful treatment-related change from normal score fluctuation (Maassen et al., 2009). However, a statistically reliable change on a CPT index—one that exceeds measurement error and would not be expected from normal score fluctuation alone—indicates only that a genuine change in cognitive task performance has occurred; it does not by itself establish that the change is clinically meaningful or reflects improvement in symptom severity or everyday functioning. Statistical reliability and clinical or functional significance therefore represent distinct questions, and CPT-based treatment monitoring should be interpreted accordingly.

In rehabilitation, CPT-derived measures may help identify relative cognitive strengths and weaknesses, establish objective baseline performance, and monitor within-person change. However, CPT profiles should not be treated as direct prescriptions for selecting particular interventions or as independent evidence of treatment efficacy, because their value for treatment matching remains unestablished (Epstein and Weiss, 2012). VR-based interventions should likewise be distinguished from VR-CPT assessment. VR platforms provide controlled and adaptable environments in which task demands and distractors can be modified, and preliminary evidence suggests possible benefits for attention and broader cognition in children with ADHD (Bashiri et al., 2017; Corrigan et al., 2023). These findings remain limited by small samples, heterogeneous interventions and outcomes, variable methodological quality, and insufficient long-term functional evidence. Rehabilitation decisions should therefore continue to integrate symptoms, developmental and cognitive characteristics, functional impairment, comorbidities, family priorities, feasibility, and observed response to intervention (Wolraich et al., 2019). Further adequately powered trials with active comparison conditions, functional outcomes, long-term follow-up, and prespecified treatment-response analyses are needed to determine whether CPT-informed or VR-based approaches provide incremental clinical benefit (Corrigan et al., 2023; Cortese et al., 2015).

## 7 Limitations of CPT

### 7.1 Low diagnostic specificity

A central limitation of CPT-based assessment is that the cognitive processes measured by these tasks are not specific to ADHD. Difficulties in sustained attention, response inhibition, response speed, and response consistency occur across a range of psychiatric, neurodevelopmental, and cognitive conditions, resulting in substantial overlap between ADHD and other clinical populations (Kofler et al., 2013; C. A. Riccio and Reynolds, 2001; Wright et al., 2014).

This transdiagnostic overlap has an important methodological corollary that is often underemphasized: much of the evidence supporting CPT sensitivity to ADHD, including the pooled sensitivity and specificity estimates reported in recent meta-analyses (e.g., AUC values generally in the 0.7–0.8 range) (Arrondo et al., 2024), derives predominantly from studies comparing children with ADHD to typically developing peers rather than to other clinical or neurodevelopmental populations. Because typically developing children are, by definition, unlikely to show elevated omission errors, commission errors, or reaction-time variability for any reason, this design tends to overestimate how well CPT indices would discriminate ADHD from the differential-diagnostic possibilities actually encountered in clinical practice, where children being evaluated for possible ADHD often present with overlapping difficulties from other conditions rather than with no attentional or behavioral difficulties at all. Diagnostic-accuracy estimates derived from ADHD-versus-typically-developing comparisons should therefore not be assumed to generalize to real-world clinical discrimination tasks.

The comorbidity between ADHD and autism spectrum disorder (ASD) illustrates this issue directly. ASD and ADHD frequently co-occur, and children with ASD—independent of any comorbid ADHD—commonly show difficulties in attentional regulation and executive function that overlap substantially with the cognitive processes CPTs are designed to measure (Lee et al., 2024; Lundervold et al., 2016). In a four-group comparison using the Conners CPT-II, children with combined ASD and ADHD showed a CPT performance profile—including a riskier response style, greater variability, and lower consistency—that more closely resembled children with ADHD alone than children with ASD alone, while intellectual functioning substantially influenced CPT performance within the ASD subgroups independent of ADHD status (Lundervold et al., 2016). A separate study directly comparing ASD, ASD with comorbid ADHD, and ADHD groups on a comprehensive attention test found that commission errors were more elevated in the ADHD and comorbid groups than in ASD alone, suggesting some specificity for response-inhibition-related indices, whereas reaction-time variability was further elevated in the comorbid group, consistent with an additive rather than ADHD-specific pattern (Lee et al., 2024). Together, these findings indicate that some CPT abnormalities attributed to ADHD may instead reflect neurocognitive features shared with ASD or amplified by their co-occurrence, rather than ADHD-specific deficits. Determining CPT specificity therefore requires comparison not only with typically developing children but with other neurodevelopmental and psychiatric populations, an evidence base that remains considerably smaller than the ADHD-versus-typically-developing literature. This gap directly supports the position taken throughout this review, and particularly in the Conclusion, that CPTs are best used as adjuncts within a broader, multimodal diagnostic process rather than as standalone or subtype-classification instruments.

CPT performance is also influenced by intellectual functioning and temporary fluctuations in alertness. Lower intellectual ability may be associated with slower responses, more omission errors, and greater response variability, while insufficient sleep may worsen attentional performance even in children without ADHD (Gruber et al., 2011; Hurford et al., 2017; Puga et al., 2019). These influences may increase the likelihood of false-positive interpretations when abnormal scores are attributed to ADHD without considering developmental, cognitive, and situational factors.

Conversely, some children with ADHD, particularly those with higher intellectual ability, may perform within normative limits despite clinically significant symptoms, contributing to false-negative interpretations (Park et al., 2011). This bidirectional overlap limits the sensitivity and specificity of individual CPT indices and helps explain their generally modest diagnostic accuracy at the individual level. Beyond this transdiagnostic overlap, the state-regulation account introduced in Section 3 offers a complementary explanation: if elevated RTV and related indices reflect moment-to-moment fluctuations in energetic state rather than a fixed trait-level deficit, a single CPT administration inherently samples only one instance of an inherently variable process, further constraining its reliability as an individual-level diagnostic marker.

### 7.2 Cross-version heterogeneity and limited comparability

CPT versions differ in task structure, stimulus modality, duration, the inter-stimulus interval (ISI) and associated event rate, target probability, response rules, and scoring conventions. These differences alter demands on sustained attention, perceptual processing, response inhibition, and motor responding, limiting direct comparisons across paradigms (Huang-Pollock et al., 2012). Stimulus characteristics can affect error rates and reaction times, while X-type and AX-type tasks may show different associations between reaction-time variability and ADHD symptoms, suggesting that similarly labeled outcomes may not reflect identical cognitive processes (Levy et al., 2018; Roebuck et al., 2016). Reaction-time variability is also measured in different ways, including raw RT standard deviation, coefficient of variation, ex-Gaussian parameters such as τ and σ, and platform-specific norm-referenced scores (Kofler et al., 2013). Because these measures differ in their dependence on mean reaction time, sensitivity to unusually slow responses, trial-exclusion procedures, score transformations, and normative frameworks, RTV scores from different CPTs should not be assumed to be numerically or psychometrically equivalent (Tamm et al., 2012). A systematic review and meta-analysis of 19 studies using commercial CPTs found only modest-to-moderate discrimination between ADHD and non-ADHD samples (Arrondo et al., 2024). Composite scores generally performed better than omission indices, whereas commission indices showed the weakest performance, with substantial variation across instruments and studies. Cross-study comparisons and interpretations based on platform-specific cutoffs should therefore consider the CPT version, task parameters, metric definitions, scoring procedures, and normative population.

### 7.3 Additional limitations specific to emerging technology-assisted CPTs

Technology-assisted CPTs face limitations beyond those affecting traditional paradigms. Large, independently replicated, age-stratified, and cross-cultural normative and validation studies remain limited, and many investigations have used small, single-site, or proof-of-concept samples (Lee et al., 2023). Although virtual-classroom CPTs detect group-level differences between individuals with ADHD and controls, variation in task design, distractors, participant characteristics, and outcomes limits comparison across systems (Goharinejad et al., 2022). Cross-cultural findings may also vary; VR-CPT classification of ADHD presentations differed between Spanish and Argentine samples, although symptom-severity differences may have contributed (Areces et al., 2025).

These paradigms depend on specific hardware, calibration procedures, sampling rates, task implementations, and analytical algorithms. Such differences may reduce cross-site reproducibility and complicate comparison between systems (Stokes et al., 2022). Eye-tracking studies have reported improved internal group classification, but findings are based mainly on individual datasets without prospective external validation. Machine-learning models have likewise shown promising internal performance, but most have relied on retrospective data and internal validation (Slobodin et al., 2020).

Current findings therefore represent preliminary evidence of potential incremental value rather than established clinical utility. Evidence also remains insufficient to determine whether selecting a technology-assisted CPT according to a child’s predominant symptom profile improves diagnosis, treatment planning, or clinical outcomes (Areces et al., 2025).

### 7.4 Additional general limitations

Beyond version- and technology-specific concerns, several limitations apply broadly across CPT paradigms. CPTs assess the cognitive domains of sustained attention, response inhibition, and response consistency—together with basic response speed—under structured conditions, but they do not comprehensively represent the broader executive and motivational heterogeneity associated with ADHD. Meta-analytic evidence indicates that executive-function difficulties extend across multiple domains and are not present uniformly in all individuals with ADHD. Motivational processes such as delay aversion may also represent partly separable pathways that are not directly captured by conventional CPT indices (Sonuga-Barke, 2002; Willcutt et al., 2005).

Ecologically oriented paradigms attempt to improve contextual representativeness by embedding CPT tasks within classroom-like settings and incorporating controlled environmental distractors. Evaluating these paradigms requires distinguishing three related but separate concepts: ecological resemblance, the extent to which a task’s surface features (a simulated classroom, background noise, visual distractors) look or sound like a real-world setting; experimental complexity, the number and sophistication of manipulated variables, sensors, or data streams a paradigm incorporates (e.g., controlled distractor conditions, motion tracking, eye tracking); and demonstrated ecological validity, direct evidence that test performance corresponds to, or predicts, behavior in real-world settings such as the classroom, home, or peer environment. A paradigm can rate highly on the first two without having established the third. Greater ecological resemblance or experimental complexity may increase sensitivity to behavior under specific, controlled distraction conditions, but neither should be equated with demonstrated ecological validity. Everyday functioning depends on changing social demands, motivation, reinforcement, self-regulation, interpersonal interactions, and environmental expectations that cannot be fully reproduced within a standardized assessment, however realistic-looking or technically complex. Consequently, performance in a realistic-looking virtual or distraction-rich setting should not automatically be assumed to predict classroom behavior, academic functioning, or impairment in daily life more accurately than performance on a traditional CPT. Establishing ecological validity requires direct evidence that test outcomes correspond to relevant real-world behavior and provide information beyond established clinical and functional measures (Cassuto et al., 2013; Neguț et al., 2017). Unless such evidence is available for a given paradigm, statements describing it as more ‘ecologically relevant’ or ‘ecologically valid’ than traditional CPTs should be qualified as reflecting ecological resemblance or experimental complexity rather than demonstrated predictive validity.

Repeated administration may also be influenced by measurement error, task familiarity, developmental change, and imperfect test–retest reliability, with these effects varying across platforms, indices, age groups, and assessment intervals. Longitudinal score changes should therefore be evaluated using version-specific reliability estimates, measurement-error parameters, and retest expectations rather than being attributed automatically to treatment response or practice effects (Zabel et al., 2009). Even after such measurement error and practice effects have been accounted for, a confirmed change in CPT performance reflects a change in cognitive task performance rather than a demonstrated change in symptom severity or real-world functioning; as discussed in Section 6.5, these represent distinct levels of outcome, and CPT-based treatment monitoring should not be assumed to substitute for direct assessment of symptomatic or functional change.

Finally, CPT performance is sensitive to temporary changes in alertness, fatigue, engagement, and motivation. Experimental sleep restriction has been associated with poorer CPT performance in both children with ADHD and typically developing controls, while reinforcement can improve performance across sustained-attention and related cognitive tasks, with the magnitude of improvement varying across individuals and groups (Fosco et al., 2015; Gruber et al., 2011). CPT scores may therefore reflect both underlying cognitive abilities and the child’s state and testing context at the time of assessment.

### 7.5 Directions for future research

Future studies should evaluate the incremental clinical value of specific CPT applications rather than infer utility from cross-sectional group differences. Multicenter, age-stratified, and cross-cultural studies should harmonize task parameters, scoring procedures, and outcome definitions and establish independently validated normative and diagnostic-performance estimates, particularly for technology-assisted CPTs (Goharinejad et al., 2022).

Prospective diagnostic-impact studies should determine whether CPT results improve diagnostic certainty, assessment efficiency, treatment decisions, functional prediction, or healthcare outcomes beyond standard assessment. Existing findings suggest that incremental value may be context-dependent: Conners’ CPT-II provided additional information when parent and teacher ratings were inconclusive, while QbTest may improve assessment efficiency when incorporated into broader clinical pathways (Tallberg et al., 2019). Multimodal models combining CPT and neurophysiological measures also require prespecified analyses and external validation because their incremental predictive value has not been established (Kim et al., 2015).

Symptom-guided selection of CPT paradigms remains an unvalidated hypothesis. It should first be examined in prospective head-to-head studies comparing symptom-guided selection with standard procedures. If reproducible incremental value is demonstrated, pragmatic diagnostic-strategy trials could then evaluate effects on clinical decision-making, assessment efficiency, treatment selection, and patient-relevant outcomes (Bellato et al., 2024; Tomlinson et al., 2025).

## 8 Conclusion

CPT paradigms in pediatric ADHD assessment can be usefully understood as occupying different positions along a continuum of clinical maturity, ranging from well-normed standardized tasks to emerging technology-assisted systems whose clinical value has yet to be established. At the more mature end of this continuum, traditional standardized CPTs—particularly the Conners CPT series and TOVA—currently offer the most established normative and psychometric foundations, while QbTest has accumulated comparatively strong evidence among technology-assisted systems and may support multimodal assessment and improved assessment efficiency when interpreted alongside routine clinical information.

Distraction-enhanced and virtual-reality paradigms, including the MOXO-dCPT and VR-CPTs, extend assessment to controlled distractors and classroom-like contexts and may provide useful information about context-dependent distractibility and behavioral regulation. However, their incremental clinical value beyond traditional CPT measures and standard assessment procedures remains uncertain. Eye-tracking-integrated, Kinect-based, and machine-learning-assisted approaches offer additional measures of gaze allocation, movement, and complex performance patterns but should currently be regarded primarily as investigational tools.

CPTs make their most distinctive contribution not as standalone diagnostic or subtype-classification instruments—a role for which their specificity remains limited—but as objective, performance-based measures that are particularly well suited to characterizing the temporal dynamics and moment-to-moment variability of attention and response control, including reaction-time variability, omission patterns, and vigilance decrement across a sustained task, features that are difficult to capture through retrospective symptom ratings or clinical interview alone. Used in this way, as objective components of comprehensive assessment rather than as standalone diagnostic instruments, their future clinical value will depend on whether specific paradigms provide meaningful information that changes diagnostic decisions, treatment selection, assessment efficiency, functional prediction, or patient outcomes. This will require standardized task and outcome definitions, direct comparisons across platforms, independently validated normative data, and prospective multicenter studies. Technological sophistication alone should not be assumed to confer clinical utility; its value must be demonstrated through rigorous and independently replicated evidence.
